# Curiosities of Brain and Nerve

**Published:** 1889-08-31

**Authors:** 


### Within the Wards.

### CURIOSITIES OF BRAIN AND NERVE.

During the last thirty years an increasing number of capable and honest physicians have devoted themselves to the study of the brain and nervous system. It will surprise the general reader to learn how many and various are the sources from which "cases" are supplied that throw light on the problems involved in the natural and extra-natural working of the brain. The nursery, the school, the hospital, the lunatic asylum, the battle-field, the physiological laboratory, and so on, all furnish their quota of living brains in various stages of operation, or of check. The human brain is a wonderful organism, and if the doctrine of evolution, as understood by physiologists and other scientists, is not merely a working hypothesis, but a principle at work, it is destined to become far more wonderful still. From the simple to the complex, from the automatic to the modifiable, from the permanently controlled to the widest freedom of voluntary action is what evolution teaches. It is curious and surely most instructive to note that philosophy, with little aid from physiology, has anticipated the latest dictum of the physiological laboratory. "Though we are compassed about with necessity," said Carlyle in 1836, "yet is the meaning of life itself freedom, voluntary force." "The passage" (in evolution), says Dr. Hughlings Jackson in 1889, "is from the most automatic to the most voluntary." No doubt Dr. Hughlings Jackson may say the world need not thank Carlyle because his generalisation was but a metaphysical formula, and not an ascertained fact, based on physical science. If there be any comfort in that Dr. Jackson is welcome to it. The fact remains that the metaphysicians in Carlyle's time, and two thousand years before that, were well aware that the highest powers of the brain were those associated with reason and will, with, in fact, "voluntary" operations. Evolution and the physiological laboratory have, therefore, not advanced either knowledge or speculation in the region of the higher brain functions. The laboratory has, however, revealed many curious facts which tend to confirm the older generalisation of the metaphysicians, and to make clear the uses of different parts of the brain, and also the intimate and inseparable connection between the senses and the cerebral functions.

Dr. Weir Mitchell records a case which is not only wonderful in itself, but which shows the extraordinary persistence of impressions made upon the brain under certain circumstances. He says: "E. C. lost his left arm, 5in. below the shoulder, nine years ago. When shot, the thumb turned into the palm, and continued in this state of spasm, so that when, six hours later, the limb was removed, the nail of the thumb had cut into the palm. He several times lifted the thumb, but it always returned to the same spot. For nine years the absent thumb still remains cutting into the palm." That is to say, although E. C.'s left arm was amputated nine years before the case was published, he still felt as if his arm and hand were parts of his body, and that the thumb was cutting into the palm. In other words, the painful impression produced on the brain by the cutting of the thumb into the palm remained for nine years. And if it remained for so long, the probability is that it will remain, more or less distinct, to the end of life. "A patient of mine," says Dr. Hughlings Jackson, "had lost one hand, but, as is most often the case, he had representing it a phantom hand. This was always in a position as if encircling a vessel. The member had been blown off when he was holding a cylinder to be charged with an explosive."

Most curious, though perfectly familiar even to medical students, is the condition known as "aphasia." As the result of injury or disease to a well-known portion of the brain, a person loses the power of articulate speech. He does not lose his voice, but only the power of articulating exactly what he wants to say. He may sometimes speak automatically, like a phonograph, but he cannot speak voluntarily, like an ordinary human being.

A signalman had had what is called a stroke of paralysis when on the railway in front of his box. As a result he entirely lost the power of voluntary speech. When asked a question of any kind he would always try to answer, but could only succeed in saying "Come on," or "Come on to me." The presumption is that he was saying, or was just going to say, these words when the fit seized him. An equally curious story is related of a woman seen by Dr. Jackson in one of the London hospitals. The poor creature had had a fall whilst laying down oil-cloth on a staircase, and had fractured her skull. Although she was almost unconscious and within a few hours of death, she kept on manipulating the counterpane of her bed exactly as she would have manipulated the oilcloth had she still been laying it down. When the nurse assured her that it was "properly laid" she would desist for a little time, and then resume her work as before. A case, as amusing as the foregoing is sad, is recorded by Dr. Savage, of a patient who firmly believed himself to be a jar of Indian pickles.

These illustrations show, among other things, the extraordinary effect upon what may be called "mental capacity" produced by very different affections and injuries of the brain. They teach all those who are anxious always to judge their fellow creatures fairly, the necessity of constantly studying individual peculiarities and mental characteristics. How many faults, and even crimes, are the almost inevitable result of excess or defect, weakness or positive disease, of certain portions of the brain, it is impossible to say. No doubt in the world a rough kind of justice is meted out by one man to another, and on the whole men may be said to receive their deserts. But how very "rough" that justice is, and how many are punished who ought to be rewarded, and rewarded who ought to be punished, who shall say? Nothing more clearly shows the necessity for a moral law, and a moral law founded on the broad and truly divine principle of always doing to others as one would wish to be done by, than the revelations of the physiological laboratory. "Put yourself in his place" is a maxim which every master and every servant, every judge and every criminal, every injured man and every injurer, should never forget. All true science is true sense, and all true sense is good morals, when things are traced to their ultimate source.

